# Rice Bran Proteins Extracted by Different Methods: Structural Properties and Antioxidant and Anti-Photoaging Activities of Their Hydrolysates

**DOI:** 10.3390/antiox15081049

**Published:** 2026-08-21

**Authors:** Xiao Wang, Hongru Liu, Wenhui Tian, Bingjie Chen, Rongshang Wang, Songheng Wu, Longshen Wang, Jinglin Zhang, Hui He, Chenxia Liu, Qiankun Wang, Chunfang Wang, Jucai Xu

**Affiliations:** 1 Crop Breeding and Cultivation Research Institution, Research Center for Agricultural Products Preservation and Processing, Shanghai Academy of Agricultural Sciences, Shanghai 201403, China; 2 School of Pharmacy and Food Engineering, Wuyi University, Jiangmen 529020, China

**Keywords:** rice bran protein extraction, microstructure, enzymatic hydrolysis, quantum chemical calculations, HaCaT photoaging

## Abstract

Differences in the composition, microstructure, infrared spectral characteristics, and enzymatic hydrolysis properties of rice bran protein extracted by four methods were investigated, and the antioxidant and anti-photoaging activities of peptides derived from the resulting hydrolysates were further evaluated. Rice bran protein obtained via ultrasonic pretreatment combined with alkaline solublilization and acid precipitation (URP) exhibited relatively high purity (59.17%) and extraction yield (47.61%), together with increased surface porosity, enhanced hydration capacity, and improved enzymatic hydrolysis performance. The URP hydrolysate (URPP) showed a protein content of 81.00%, a degree of hydrolysis of 33.57%, and marked antioxidant activity (ABTS, 684.21; ORAC, 2016.15 μmol TE/g sample). The identified peptides were predominantly short and enriched in hydrophobic amino acids. Structural analysis suggested that the indole N-H group of tryptophan may play an important role in the antioxidant activity of these peptides. Moreover, these peptides alleviated UVB-induced photoaging in HaCaT cells by reducing oxidative stress and inflammatory responses and downregulating the mRNA expressions of AP-1, MMP-1 and MMP-3. Overall, these findings reveal an association between the extraction method, structural characteristics, and enzymatic hydrolysis properties of rice bran protein and the biological activities of its derived peptides, providing a basis for the high-value utilization of rice bran protein and the development of antioxidant and anti-photoaging functional ingredients.

## 1. Introduction

China’s annual rice production is approximately 185 million tons, generating about 10 million tons of rice bran, accounting for 5–7% of total rice output [1]. However, the utilization efficiency of rice processing residues remain low, as reflected by limited utilization rates, insufficient product diversity, and low added value. Currently, only 10–15% of rice bran is utilized for oil extraction or the production of high-value products, such as calcium phytate, inositol, and oryzanol, whereas the majority is used as animal feed or discarded, resulting in substantial resource waste [2]. Rice bran contains approximately 12–15% protein and exhibits favorable nutritional and health-related properties [3]. Its protein composition is well balanced, with a protein efficiency ratio (PER) of 1.6–1.9, approaching that of casein (2.5), and high digestibility [4]. In addition, rice bran protein possesses a balanced amino acid profile and potential bioactivities, making it a valuable plant protein resource. However, its poor solubility limits its application in food and functional product development [5]. Therefore, enzymatic hydrolysis and bioactive peptide extraction strategies provide an effective approach for improving the functional properties and high-value utilization of rice bran protein.

In recent years, the preparation of functional bioactive peptides via proteolytic hydrolysis of rice bran protein has received increasing research attention [6]. This approach improves protein solubility and confers diverse biological activities on the resulting peptides, including antihypertensive effects, fatigue resistance, and promotion of alcohol metabolism [5]. Among these activities, antioxidant capacity is considered a fundamental basis for multiple health benefits, with amino acid residues enriched in tyrosine (Tyr) and tryptophan (Trp), which possess strong antioxidant properties, regarded as key structural determinants [7]. It has been reported that rice protein contains 6.49% total Tyr and Trp, compared with 5.94% in casein, and both proteins are recognized as excellent sources for the preparation of antioxidant peptides. Moreover, several peptide fragments enriched in aromatic amino acids, such as Tyr, Trp, and phenylalanine (Phe), have been identified in rice protein hydrolysates and exhibit significantly enhanced antioxidant activity [7]. Therefore, from the perspectives of protein structural characteristics and enzymatic behavior, the systematic and rational development of rice bran protein is of considerable practical significance for the high-value utilization of rice bran resources.

Different protein extraction methods have been shown to markedly affect protein structure, enzymatic properties, and antioxidant activity. Compared with other approaches, alkali solubilization–acid precipitation has been reported to induce protein denaturation and aggregation, resulting in reduced solubility. Enzymatic extraction is generally conducted under mild conditions and better preserves the native protein conformation [6]; however, extraction yields are often limited. In recent years, combined technologies incorporating ultrasound, microwave, high-pressure, or subcritical water treatments have been increasingly applied. These strategies promote protein unfolding, thereby significantly improving extraction efficiency and antioxidant activity. For example, multistage ultrasonic-assisted alkaline extraction followed by acid precipitation increased rice bran protein yield from 38.9% to 65.7%, while optimizing secondary structure composition, solubility, and digestibility [6]. In addition, proteins obtained by ultrasonic-assisted extraction exhibited higher trolox equivalent antioxidant capacity (TEAC) values and enhanced DPPH radical scavenging activity [6]. Structural alterations in proteins directly affect enzyme recognition and peptide bond cleavage, with moderate unfolding facilitating the production of low-molecular-weight peptides with improved bioactivity. Increased exposure of structural regions enriched in antioxidant amino acid residues, such as Tyr and Trp, further promotes the formation of antioxidant peptides [7]. Moreover, antioxidant activity can be indirectly enhanced through modulation of protein surface hydrophobicity and solubility during extraction. For instance, optimization of the enzymatic hydrolysis process of jasmine rice bran protein resulted in high antioxidant capacity and abundant low-molecular-weight peptides [8]. Consequently, the rational selection of extraction techniques enables the optimization of structural and functional properties of rice bran protein, providing a theoretical basis and technical support for its high-value application in functional foods. Furthermore, the integration of quantum chemical calculations and human epidermal keratinocytes (HaCaT cells) models is expected to facilitate the elucidation of antioxidant and anti-photoaging mechanisms of rice bran protein-derived peptides, thereby guiding targeted enzymatic hydrolysis and functional development.

This study prepared rice bran proteins using four extraction strategies: ASAP, ultrasonic pretreatment followed by ASAP, enzymatic pretreatment followed by ASAP, and combined ultrasonic and enzymatic pretreatment followed by ASAP, yielding RP, URP, ERP, and UERP, respectively. The nutritional composition, microstructure, chemical structure, and enzymatic hydrolysis properties of the four proteins were comparatively evaluated. RP and URP were further hydrolyzed to obtain RPP and URPP, respectively, followed by characterization of their peptide profiles and antioxidant activities. Quantum chemical calculations and an UVB-induced HaCaT cell model were used to investigate the structural basis of peptide antioxidant activity and their protective effects against oxidative stress, inflammation, and photoaging. Collectively, this study aimed to establish the relationship between extraction strategy, protein structure, enzymatic hydrolysis characteristics, and the antioxidant and anti-photoaging activities of derived peptides, thereby providing a basis for the targeted preparation and high-value utilization of rice bran protein and the development of functional peptides.

## 2. Materials and Methods

### 2.1. Materials

Rice bran samples of the Nanjing 46 variety were provided by Shanghai Shengzhi Agricultural Products Co., Ltd (Shanghai, China). After collection in 2024, the samples were immediately dried at 45 °C and stored at −20 °C under sealed and dark conditions. Hexane, sodium hydroxide, hydrochloric acid, α-amylase, and cellulase were obtained from Beijing Yishan Huitong Technology Co., Ltd (Beijing China). Trolox (6-hydroxy-2,5,7,8-tetramethylxanthene-2-carboxylic acid) and 2,2′-azobis(2-methylpropionamide) dihydrochloride (AAPH) were purchased from the same supplier. 2,2′-Azino-bis(3-ethylbenzothiazoline-6-sulfonic acid) diammonium salt (ABTS) and sodium fluorescein were obtained from Beijing Zhongcheng Jinnian Technology Co., Ltd (Beijing China). Synthetic peptides (WGWAF, WGRF, MRF, WGGWLR, RPF, HMFGF, VFF, MCFLPL, SGF, WGRLL, WPF, FGF, WWYF, LFF, GGF, WL, RF, FGAF, LW, and FPPP) with purities exceeding 95% were obtained from Nanjing JiePeptide Biotechnology Co., Ltd (Nanjing China). Solvents for UPLC analysis were of LC-MS grade and were supplied by Sigma-Aldrich (St. Louis, MO, USA). Deionized water was prepared using a Milli-Q water purification system (Millipore, Bedford, MA, USA). HaCaT cells and complete culture medium were obtained from Shanghai Fuheng Biotechnology Co., Ltd. (Shanghai, China). Fetal bovine serum and penicillin streptomycin were obtained from Life Technologies (Carlsbad, CA, USA). Assay kits for malondialdehyde (MDA) content, glutathione (GSH) level, superoxide dismutase (SOD) activity, and CCK-8 cell viability were obtained from Nanjing Jiancheng Bioengineering Institute (Nanjing, China). Enzyme-linked immunosorbent assay kits for IL-6, IL-1β, and TNF-α were purchased from Xinbosheng Biotechnology Co., Ltd. (Shenzhen, China). All other chemical reagents were of analytical grade.

### 2.2. Extraction of Rice Bran Protein

#### 2.2.1. Degreasing Treatment of Rice Bran

Rice bran was ground and sieved through an 80-mesh sieve. n-Hexane was added at a ratio of 1:10 (*w*/*v*) and the mixture was stirred magnetically for 12 h. The upper n-hexane layer was removed, and the residue was allowed to volatilize residual n-hexane in a fume hood, followed by drying at 45 °C for 24 h to obtain degreased rice bran [9].

#### 2.2.2. Extraction of Rice Bran Protein by ASAP

Rice bran protein was extracted from defatted rice bran using the ASAP method at a material-to-liquid ratio of 1:10 (*w*/*v*). The suspension was adjusted to pH 11.5 with 1.0 M NaOH, stirred at 45 °C for 1 h, and centrifuged (8000× *g*, 15 min, 4 °C). The supernatant was adjusted to pH 5.5 with 1.0 M HCl for isoelectric precipitation, and the precipitate was freeze-dried and designated as RP [10].

For modified extraction, ultrasonic (URP), enzymatic (ERP), and combined ultrasonic–enzymatic (UERP) pretreatments were applied. Ultrasonic treatment was performed at 1500 W and 30 °C for 1 h with a 15 min on/10 min off cycle [11]. Enzymatic treatment was conducted with α-amylase (Sigma-Aldrich, A3306, 40,000 U/mL) and cellulase (Sigma-Aldrich, C2730, 700 U/g) at 0.2 g/L each, pH 5.5 and 50 °C for 2 h [12]. For UERP, the samples were first subjected to ultrasonic treatment and subsequently treated with both enzymes under the same enzymatic conditions for 2 h [13].

### 2.3. Determination of the Composition and Extraction Yield of Rice Bran Protein

The proximate composition of rice bran was analyzed using established standard procedures. Nitrogen content was measured with a vario EL cube elemental analyzer (Elementar Analysensysteme GmbH, Langenselbold, Germany), and protein content was estimated using a nitrogen-to-protein conversion factor of 5.95. Lipid content was obtained by Soxhlet extraction, whereas ash, crude fiber, starch, and moisture were determined according to GB 5009.4-2016, GB/T 5009.10-2003, GB 5009.9-2016, and GB 5009.3-2016, respectively [14]. Protein extraction yield was expressed as the percentage of protein recovered from the raw material relative to its initial protein content, as calculated using the following equation:

$$\mathrm{Protein}\;\mathrm{extraction}\;\mathrm{yield}\;(\%)\;=\;\frac{m_{\mathrm{extract}}\;\times \;C_{\mathrm{extract}}}{m_{\mathrm{raw}}\;\times \;C_{\mathrm{raw}}}\times 100$$

where m_extract_ is the mass of the protein extract (g); C_extract_ is the protein content of the extract (%); m_raw_ is the mass of the raw material (g); and C_raw_ is the protein content of the raw material (%).

### 2.4. Scanning Electron Microscopy (SEM) Analysis

Rice bran protein samples were mounted on conductive adhesive tape and fixed onto sample stubs. The samples were sputter-coated with a thin layer of gold prior to observation. Surface morphology was examined using a scanning electron microscope (SU8010, Hitachi, Tokyo, Japan) under the following operating conditions: accelerating voltage of 5 kV, beam current of 50 pA, and magnification of 500× and 1000× [15].

### 2.5. Fourier Transform Infrared (FTIR) Spectroscopy Analysis

Rice bran protein (2 mg) was thoroughly mixed with potassium bromide (KBr) and ground into a homogeneous mixture. The mixture was compressed into transparent pellets using a hydraulic press. FTIR spectra were recorded using a Nicolet iS50 FTIR spectrometer (Thermo Fisher Scientific, Waltham, MA, USA) over the range of 4000–400 cm^−1^ at a resolution of 2 cm^−1^ with 32 scans. KBr was used as the background for baseline correction [16].

### 2.6. Enzymatic Hydrolysis of Rice Bran Proteins

Enzymatic hydrolysis of the four rice bran protein samples was performed using alcalase (2.4 AU/g; Novozymes A/S, Bagsværd, Denmark) under its optimal conditions (pH 8.0, 55 °C) [17]. Each protein sample (5 g) was dispersed in 100 mL of distilled water, and the pH was adjusted to 8.0. The enzyme was added at 4% (*w*/*w*) based on the substrate protein. Hydrolysis was carried out at 55 °C for 4 h under the optimal pH. The reaction was terminated by heating at 100 °C for 10 min, followed by cooling to room temperature. The hydrolysate was centrifuged, and the supernatant was collected and lyophilized. The resulting peptides were designated as RPP, URPP, ERPP, and UERPP, corresponding to the respective protein samples.

### 2.7. Determination of the Degree of Hydrolysis (DH)

The DH was determined using the o-phthaldialdehyde (OPA) method, following Wang et al. [17] with minor modifications. The OPA reagent (200 mL) was prepared as follows: 160.0 mg of OPA was dissolved in 4.0 mL of ethanol. Separately, 7.62 g of borax was dissolved in approximately 150 mL of deionized water, and 200.0 mg of sodium dodecyl sulfate (SDS) and 176 mg of dithiothreitol (DTT) were added. The two solutions were then combined and diluted with deionized water to a final volume of 200 mL.

For the assay, 20 μL of the sample solution was mixed with 150 μL of OPA reagent and incubated at room temperature for 2 min. The absorbance was measured at 340 nm using a microplate reader (Infinite M200 PRO, Tecan, Männedorf, Switzerland). Deionized water was used as a blank (A_blank_), and a 0.1 mg/mL serine solution served as the standard (A_standard_). The serine NH_2_ equivalent (Ser NH_2_) and DH were calculated using the following equations:

$${SerNH}_{2}(mmol/g)=\frac{A_{sample}-A_{blank}}{A_{standard}-A_{blank}}\times 0.9516\times C_{sample}$$

$$DH(\%)=\frac{Ser{NH}_{2}-\beta }{\alpha h_{tot}}\times 100\%$$

where C_sample_ (g/L) represents the protein concentration of the sample. The constants α, β, and htot were 1.0, 0.4, and 8.6 mmol/g, respectively. The 0.1 mg/mL serine standard corresponds to a molar concentration of 0.9516 mM.

### 2.8. Determination of Protein Recovery Rate

The protein contents of freeze-dried rice bran protein hydrolysates and their corresponding untreated rice bran proteins were determined using an elemental analyzer (Elementar Analysensysteme GmbH, Langenselbold, Germany). Protein recovery rate was calculated as the ratio of total protein in hydrolysate to that in the untreated rice bran protein prior to hydrolysis, following the method of Wang et al. [18].

### 2.9. Antioxidant Activity Assays

#### 2.9.1. ABTS Radical Scavenging Activity

The ABTS radical scavenging activity was determined using a modified method based on Wang et al. [18]. ABTS (7 mM) was mixed with potassium persulfate (2.45 mM) in equal volumes and incubated in the dark at room temperature for 12–16 h to generate the ABTS•^+^ stock solution. Before analysis, the solution was diluted with 50 mM phosphate buffer (pH 7.4) to an absorbance of 0.700 ± 0.020 at 734 nm. For the assay, 150 μL of the diluted ABTS•^+^ solution was mixed with 50 μL of the sample and incubated at 30 °C for 30 min. The absorbance was measured at 734 nm using an Infinite M200 PRO microplate reader (Tecan, Männedorf, Switzerland). Phosphate buffer served as the control, and Trolox was used as the reference standard. Antioxidant capacity was expressed as TEAC. The hydrolysate was tested at 125 μg/mL, whereas the synthetic peptides were tested at 0.1 mM.

For the ABTS assay, a Trolox calibration curve was established over a concentration range of 12.5–100 μM, corresponding to a scavenging activity range of 5.38–66.64%, and was described by the equation y = 0.7086x − 4.7739.

#### 2.9.2. Oxygen Radical Absorbance Capacity (ORAC) Assay

The ORAC assay was performed using a modified method based on Wang et al. [17] and Ren et al. [19]. Briefly, 20 μL of the sample was mixed with 120 μL of fluorescein solution (117 nM) and pre-incubated at 37 °C for 15 min in the dark. The reaction was initiated by adding 60 μL of AAPH solution (40 mM). Fluorescence intensity was recorded at 1 min intervals for 100 min using a microplate reader (Infinite M200 PRO, Tecan, Männedorf, Switzerland), with excitation and emission wavelengths set at 485 nm and 520 nm, respectively. A 75 mM phosphate buffer (pH 7.4) was used as the blank, and Trolox was employed for calibration. Both fluorescein and AAPH were prepared using the same phosphate buffer. The ORAC values were calculated based on the net area under the fluorescence decay curve (Net AUC) and expressed as Trolox equivalents. The hydrolysate was tested at 125 μg/mL, whereas the synthetic peptides were tested at 0.1 mM.

For the ORAC assay, the Trolox calibration curve was established over a concentration range of 0–100 μM, corresponding to a Net AUC range of 0–70.53, and was described by the equation y = 1.2056x + 2.6956.

### 2.10. Identification of Protein-Derived Peptides

#### 2.10.1. Ultrafiltration Fractionation

Following the procedure described by Wang et al. with minor modifications, the enzymatic hydrolysate was dissolved in deionized water at 50 mg/mL and filtered through a 0.45 μm membrane [17]. The filtrate was sequentially fractionated using centrifugal ultrafiltration devices with molecular weight cut-offs (MWCOs) of 100, 30, 10, and 3 kDa, yielding five fractions: P1 (>100 kDa), P2 (30–100 kDa), P3 (10–30 kDa), P4 (3–10 kDa), and P5 (<3 kDa). The 3 kDa fractionation was performed using a Macrosep/Jumbosep centrifugal ultrafiltration device equipped with an Omega™ modified polyethersulfone membrane (Pall Corporation, New York, NY, USA; maximum capacity, 5 mL; maximum relative centrifugal force, 7000× *g*; operating temperature, 0–40 °C; nominal recovery, >90%) for 30 min.

#### 2.10.2. Mass Spectrometry Identification

Peptide sequences were identified using a UPLC-Q-TOF-MS/MS system (Triple TOF 5600+ LC MS system, AB SCIEX, Framinghan, MA, USA). Chromatographic separation was performed on an ACQUITY UPLC HSS T3 column maintained at 45 °C. Mobile phase A was water containing 0.1% (*v*/*v*) formic acid, and mobile phase B consisted of 80% acetonitrile. The flow rate was set at 0.4 mL/min. The gradient elution program was as follows: 0–2.0 min, 92.0% A; 2.0–45.0 min, 92.0–72.0% A; 45.0–55.0 min, 72.0–60.0% A; 55.0–56.0 min, 60.0–5.0% A; and 56.0–66.0 min, 5.0% A. Electrospray ionization (ESI) was operated in positive ion mode, and data was acquired using the MSE scan mode over a mass range of *m*/*z* 50–2000. The obtained MS/MS data were processed and analyzed using the Mascot database [20].

### 2.11. Screening of Antioxidant Peptides

The unique peptides identified in RPP and URPP were screened for potential allergenicity and toxicity using AllerTOP and ToxinPred, respectively, and their potential bioactivity was assessed using PeptideRanker [17]. Peptides predicted to be allergenic or toxic were excluded, and the remaining candidates were ranked according to their PeptideRanker scores. The top 10 peptides from each sample were selected for synthesis and subsequent evaluation of antioxidant activity.

### 2.12. Density Functional Theory (DFT) Calculations

The initial three-dimensional structure of the selected antioxidant peptide was constructed using Chem3D 20.1 software (PerkinElmer Informatics, Waltham, MA, USA). Geometry optimization was performed at the M06-2X/6-31G(d) level with the Gaussian 09 software package (Revision D.01,Gaussian, Inc., Wallingford, CT, USA). Frequency analysis was subsequently carried out to confirm that the optimized structure corresponded to a true minimum on the potential energy surface, as indicated by the absence of imaginary frequencies. The optimized geometry was then used to calculate electronic properties, including the Mulliken charge distribution and bond length characteristics [21].

### 2.13. In Vitro Anti-Photoaging Activity Evaluation

#### 2.13.1. HaCaT Cell Culture

HaCaT cells were maintained in a humidified incubator at 37 °C with 5% CO_2_. The culture medium was Dulbecco’s modified Eagle’s medium (DMEM) supplemented with 10% fetal bovine serum and 1% penicillin streptomycin. The medium was replaced every 2 days [22].

#### 2.13.2. Cell Viability Assessment

The cytotoxicity of peptides was evaluated using the MTT assay. HaCaT cells were seeded into 96-well plates at 1 × 10^4^ cells/well and cultured for 24 h, followed by treatment with peptides at concentrations ranging from 0.01 to 800 μM for another 24 h. A control group without peptide treatment and a vehicle control group containing the corresponding solvent were included. After incubation with MTT solution (5 mg/mL) for 4 h at 37 °C in the dark, the formazan crystals were dissolved in DMSO, and absorbance was measured at 570 nm to determine cell viability.

The cytoprotective effects of selected peptides (WGRF, WPF, WL, and LW) against UVB-induced cellular damage were evaluated using the CCK-8 assay [22]. HaCaT cells were seeded into 96-well plates at 1 × 10^4^ cells/well and cultured for 24 h. Except for the control group, cells were irradiated with a G8T5E UVB-313 nm lamp (8 W, peak wavelength: 313 nm) at a distance of 5 cm. The irradiance at the cell surface was 1.3 mW/cm^2^, and UVB exposure for 100 s resulted in a total dose of 130 mJ/cm^2^. Following irradiation, cells were treated with WGRF, WPF, WL, or LW (100 μM), or GSH (5 mM, positive control), and incubated for 24 h. Cell viability was subsequently determined by adding CCK-8 reagent and measuring absorbance at 490 nm. The absorbance was measured using an Infinite M200 PRO microplate reader (Tecan, Männedorf, Switzerland). Cell viability was calculated as follows:

Cell viability (%) = (OD_2_ − OD_1_) × 100/(OD_1_ − OD_0_)

where OD_0_ represents the absorbance of cell-free background wells, OD_1_ represents the absorbance of the normal control group, and OD_2_ represents the absorbance of the corresponding treatment group. For the synthetic peptide cytotoxicity assay, OD_2_ corresponded to the groups treated with 0.01–800 μM peptide (0.01, 0.1, 1, 5, 10, 50, 100, 200, 400, and 800 μM). For the UVB-induced oxidative damage assay, OD_2_ corresponded to the UVB model, UVB + 100 μM candidate peptide, and UVB + 5 mM GSH groups.

#### 2.13.3. Intracellular Reactive Oxygen Species Detection

Logarithmically growing HaCaT cells were enzymatically dissociated and resuspended in complete culture medium. Cells were seeded into 48-well plates at a density of 2 × 10^4^ cells per well in 300 μL of medium and incubated at 37 °C with 5% CO_2_ for 24 h. Four experimental groups were established: control (no UVB), UVB model, positive control (5 mM GSH), and peptide-treated (100 μM). Except for the control, cells were exposed to UVB irradiation for 100 s, followed by treatment with GSH or peptides for 24 h. After treatment, cells were incubated with 1 μg/mL DCFH DA at 37 °C in the dark for 30 min, washed three times with phosphate-buffered saline, and observed under an inverted fluorescence microscope [23]. Fluorescence intensity was quantified using ImageJ software (v1.54), and intracellular reactive oxygen species (ROS) levels were calculated as follows: ROS level (%) = (fluorescence intensity of treated group/fluorescence intensity of control group) × 100 [23].

#### 2.13.4. MDA, GSH, SOD, and Cytokine Measurements

HaCaT cells were seeded into 6-cm dishes at a density of 5 × 10^5^ cells per dish in 3 mL of complete medium and incubated at 37 °C with 5% CO_2_ for 24 h. Experimental grouping and treatments were performed as previously described. After UVB irradiation, cells were treated with GSH or peptides and incubated for 24 h. Cells and culture supernatants were then collected. Cell pellets were resuspended in pre-chilled extraction buffer (Shanghai Yuanye Bio-Technology, Shanghai, Chian, Cat. No. R32687) at 5 × 10^6^ cells/mL, and lysed by ultrasonication (200 W, 3 s on/3 s off pulses, total 3 min), followed by centrifugation at 12,000× *g* for 10 min. MDA levels were determined using a commercial assay kit, while GSH content and SOD activity were measured using the corresponding assay kits [24]. Supernatants were centrifuged at 1000× *g* for 5 min, and IL-6, IL-1β, and TNF-α levels were quantified using enzyme-linked immunosorbent assay (ELISA) kits [24]. Absorbance measurements were performed using an Infinite M200 PRO microplate reader (Tecan, Männedorf, Switzerland).

#### 2.13.5. Effects on AP-1, MMP-1, and MMP-3 Gene Expression

Cell grouping and treatments were performed as described above. Total RNA was extracted using the TRI-RCP Kit (Ribolab Biotechnology Co., Qingdao, China), and cDNA was synthesized with the PrimeScript™ RT Kit (RRO37A, TaKaRa, Shiga, Japan). Quantitative real-time PCR was carried out on a LightCycler 96 system (Roche, Basel, Switzerland). The amplification protocol consisted of an initial denaturation at 95 °C for 30 s, followed by 40 cycles of 95 °C for denaturation and 65 °C for annealing. Glyceraldehyde-3-phosphate dehydrogenase (GAPDH) was used as the internal reference gene. Relative mRNA expression levels of AP-1, MMP-1, and MMP-3 were calculated using the 2^−ΔΔCt^ method. Primer sequences are listed in Table S1 [25].

### 2.14. Statistical Analysis

Data are presented as the mean ± standard deviation (SD) from three independent experiments. Statistical analyses were performed using GraphPad Prism 6 (GraphPad Software, San Diego, CA, USA). Data normality and homogeneity of variance were assessed before analysis. Differences among groups were evaluated using one-way analysis of variance (ANOVA), followed by Dunnett’s multiple comparisons test for comparisons with the control group. A *p*-value < 0.05 was considered statistically significant. Statistical significance is indicated as *p* < 0.05, *p* < 0.01, *p* < 0.001, and *p* < 0.0001.

## 3. Results and Discussion

### 3.1. Proximate Composition of Raw Rice Bran and Rice Bran Protein Samples

Rice bran, a major byproduct of rice milling, contains substantial amounts of fat and crude fiber accumulated during husking and polishing [26]. The proximate composition of raw rice bran and its protein samples is summarized in Table 1. RP obtained by the conventional ASAP method exhibited relatively high protein purity (~65.83%), but the extraction yield was relatively low (~37.34%). Protein extraction efficiency was significantly improved by ultrasonication or enzymatic hydrolysis, and their combined application further increased the yield to ~50.70%, albeit with a concomitant decrease in protein purity (~37.83%). Notably, integration of ultrasonic treatment with the ASAP method achieved a balanced enhancement of both protein purity (~59.17%) and extraction yield (~47.61%), consistent with previous reports. For example, Saydul et al. [6] reported that two-stage ultrasonication during ASAP significantly increased rice bran protein yield to 65.7%, compared with ~38.9% obtained using conventional alkaline extraction, while maintaining desirable protein purity and functional properties. Similarly, Anyiam et al. [27] demonstrated that co-extraction strategies combining ultrasonic and enzymatic treatments improved both protein yield and purity relative to traditional alkali precipitation methods. Collectively, these findings indicate that assisted extraction approaches can effectively enhance protein recovery from rice bran.

### 3.2. Scanning Electron Microscopy Analysis of Rice Bran Protein

**Figure 1:**
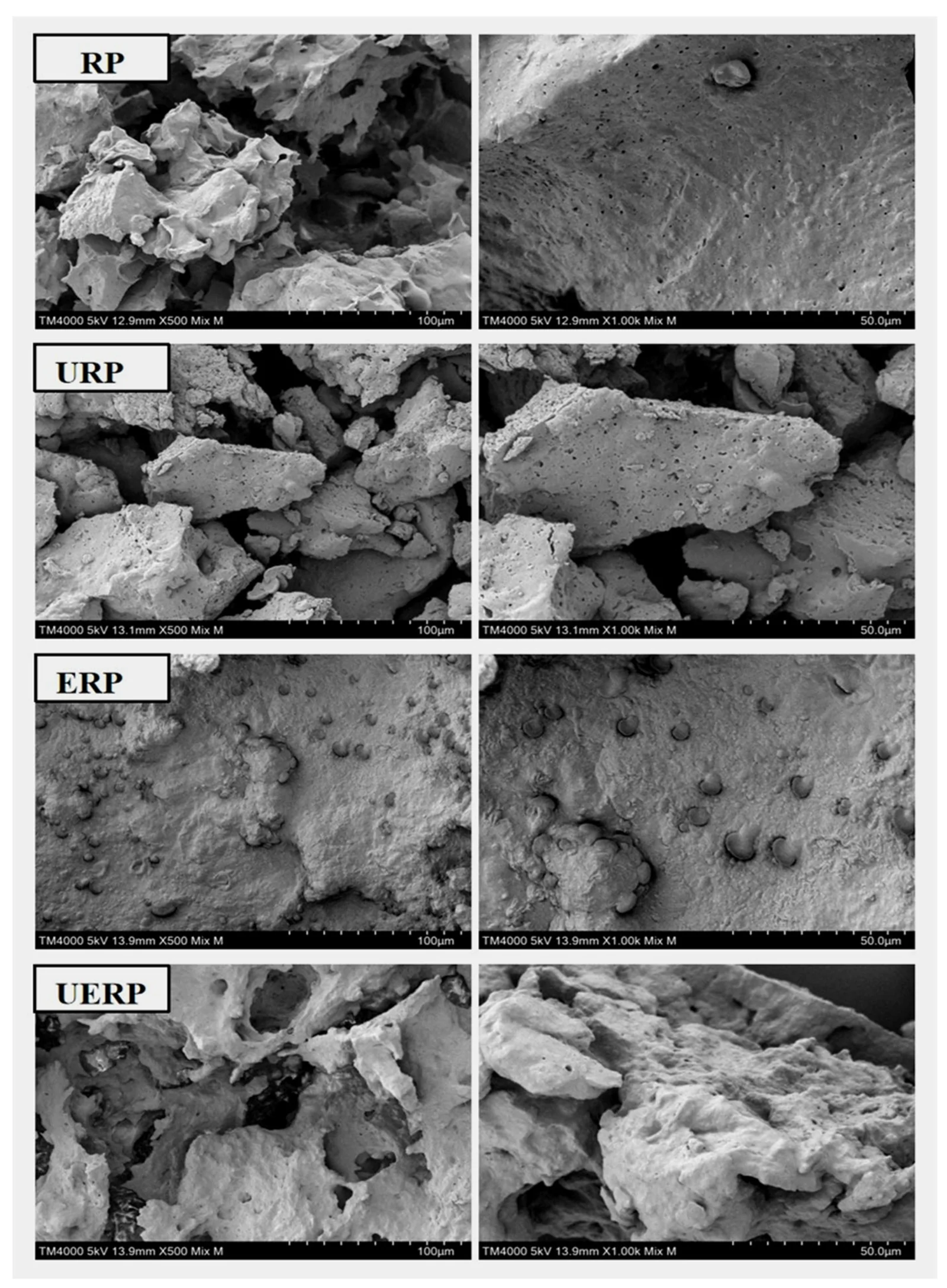
SEM images of rice bran protein samples obtained using different extraction methods (RP, URP, ERP, and UERP) at 500× and 1000× magnifications. Similar morphologies at different magnifications are due to the use of the same sample.

The microstructural characteristics of rice bran protein are presented in Figure 1. Protein extracted using the conventional ASAP method (RP) exhibited a relatively smooth surface with a loosely arranged, multilayered lamellar structure, which may be attributed to the solvent-induced precipitation. Following ultrasonic treatment, the microstructure of URP was markedly altered. Enlarged surface pores and disrupted structural organization were observed, consistent with cavitation-induced mechanical effects. Ultrasonication has also promoted lipid–protein interactions, leading to protein coagulation and aggregation, and consequently a more complex and irregular morphology. Rice bran protein obtained after enzymatic pretreatment (ERP) showed an increased starch content, potentially due to the disruption of cellulose–starch interactions and the release of bound starch. Enzymatic hydrolysis may also generate smaller starch fractions with greater solubility. SEM images revealed relatively compact and aggregated ERP particles with abundant surface-associated materials, possibly related to starch redistribution. Following combined ultrasonic and enzymatic pretreatment, the resulting protein (UERP) exhibited higher lipid and starch contents, together with a more irregular and rough surface. These surface features may reflect the redistribution or aggregation of non-protein components, such as lipids and starch. Overall, ultrasonic–enzymatic pretreatment may modify the microstructure and component distribution of rice bran, thereby potentially influencing protein extraction efficiency and functional properties [28].

### 3.3. FTIR Spectroscopy Analysis

**Figure 2:**
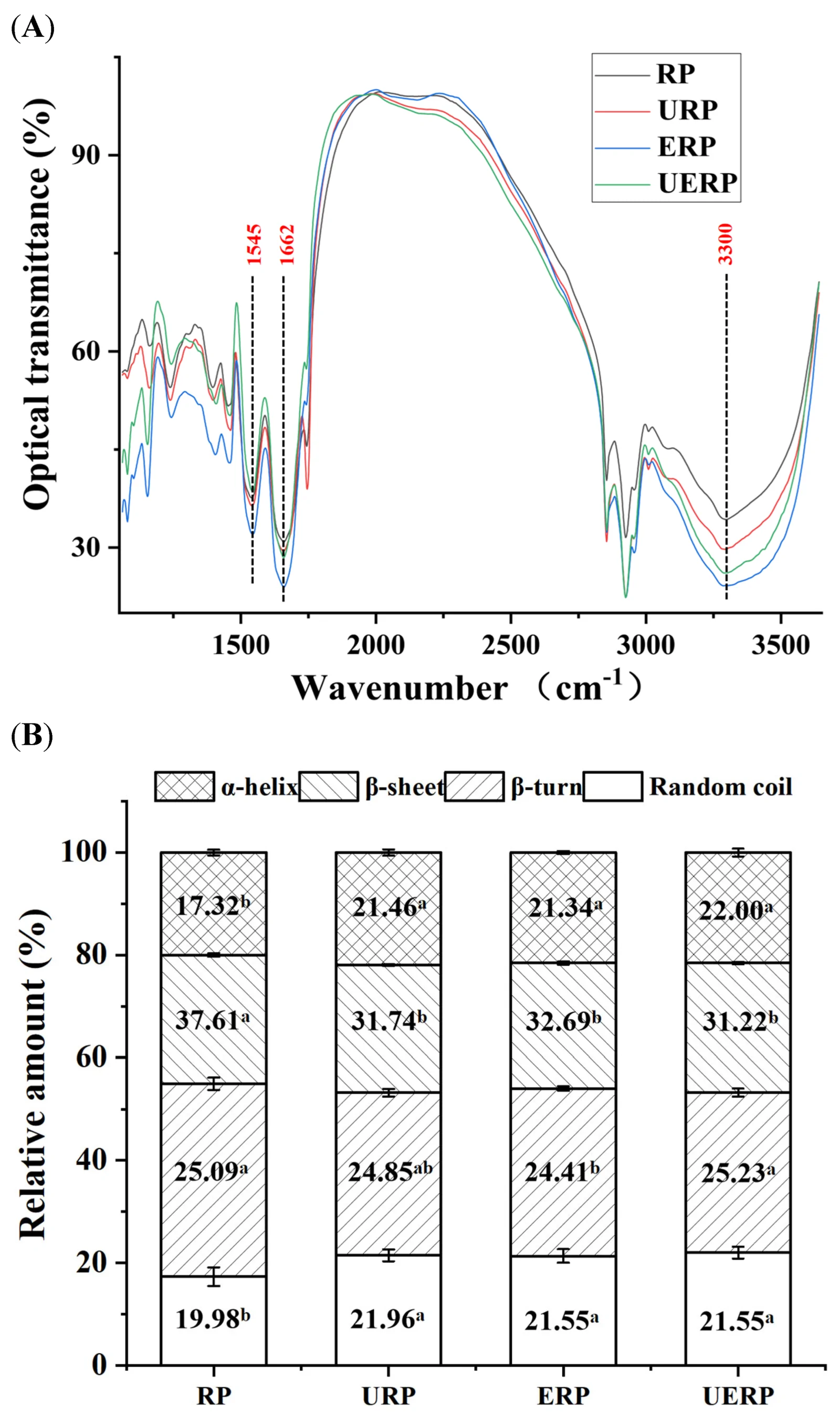
FTIR spectra (**A**) and secondary structure content (**B**) of rice bran protein samples obtained by different extraction methods. Data are presented as mean ± SD (*n* = 3). Different lowercase letters (a–b) indicate significant differences among groups (*p* < 0.05) according to one-way analysis of variance (ANOVA) followed by Dunnett’s multiple comparison test.

Infrared spectroscopy was employed to monitor structural changes in proteins, particularly secondary structure variations reflected by shifts in the Amide I (1600–1700 cm^−1^) and Amide II (1500–1600 cm^−1^) bands. The Amide I band, primarily associated with C=O stretching vibrations, is widely regarded as a key region for secondary structure analysis, whereas the Amide II band, arising from N-H bending and C-N stretching vibrations, serves as a supplementary indicator. The FTIR spectrum of rice bran protein is shown in Figure 2. The Amide I and Amide II bands were observed at approximately 1662 cm^−1^ and 1545 cm^−1^, respectively. In addition, a broad absorption band in the range of 3750–3000 cm^−1^, with a prominent peak around 3300 cm^−1^, was attributed to N-H and O-H stretching vibrations, indicating the presence of amino and hydroxyl groups. Peak-Fit software was used to deconvolute the Amide I region (1600–1700 cm^−1^) for quantitative analysis of the secondary structure composition. Rice bran protein RP predominantly consisted of β-sheet structures (~37.61%), followed by β-turns (~25.09%). In contrast, URP, ERP, and UERP samples exhibited reduced β-sheet content (31.22–32.69%), accompanied by an increase α-helix content (21.34–22.00%) and random coil proportion (21.55–21.96%). The transition from β-sheet to α-helix and random coil structures suggested enhanced protein flexibility, which may influence stability, functionality, and bioactivity. These findings are consistent with previous reports indicating that physical and enzymatic treatments can induce conformational changes in rice bran proteins, thereby affecting their functional characteristics [29,30].

### 3.4. Antioxidant Activity and Degree of Hydrolysis of Rice Peptides

The antioxidant activity of rice bran protein peptides is presented in Table 2. Peptides derived from ultrasonically treated rice bran protein (URPP) exhibited the highest antioxidant capacity among all treatment groups. Specifically, URPP showed an ORAC value of 2016.15 ± 6.22 μmol TE/g sample and an ABTS radical scavenging capacity of 684.21 ± 17.67 μmol TE/g sample. This enhanced antioxidant activity may be attributed to structural modifications induced by ultrasonication. The loosened structure of URP facilitates enzymatic hydrolysis, leading to the generation of a higher quantity of bioactive peptides with improved free radical scavenging capacity. In comparison, rice bran protein peptides obtained via conventional alkali extraction (RPP) also exhibited considerable antioxidant activity. However, their ORAC and ABTS values were slightly lower than those of URPP. This difference is likely due to the more compact structure of RPP, which limits enzymatic accessibility and peptide release. The comparable antioxidant activity observed for ERP and UERP may be related to their relatively lower protein content, which restricts the generation of antioxidant peptides [6].

### 3.5. Sequence Analysis of Rice Peptides

**Figure 3:**
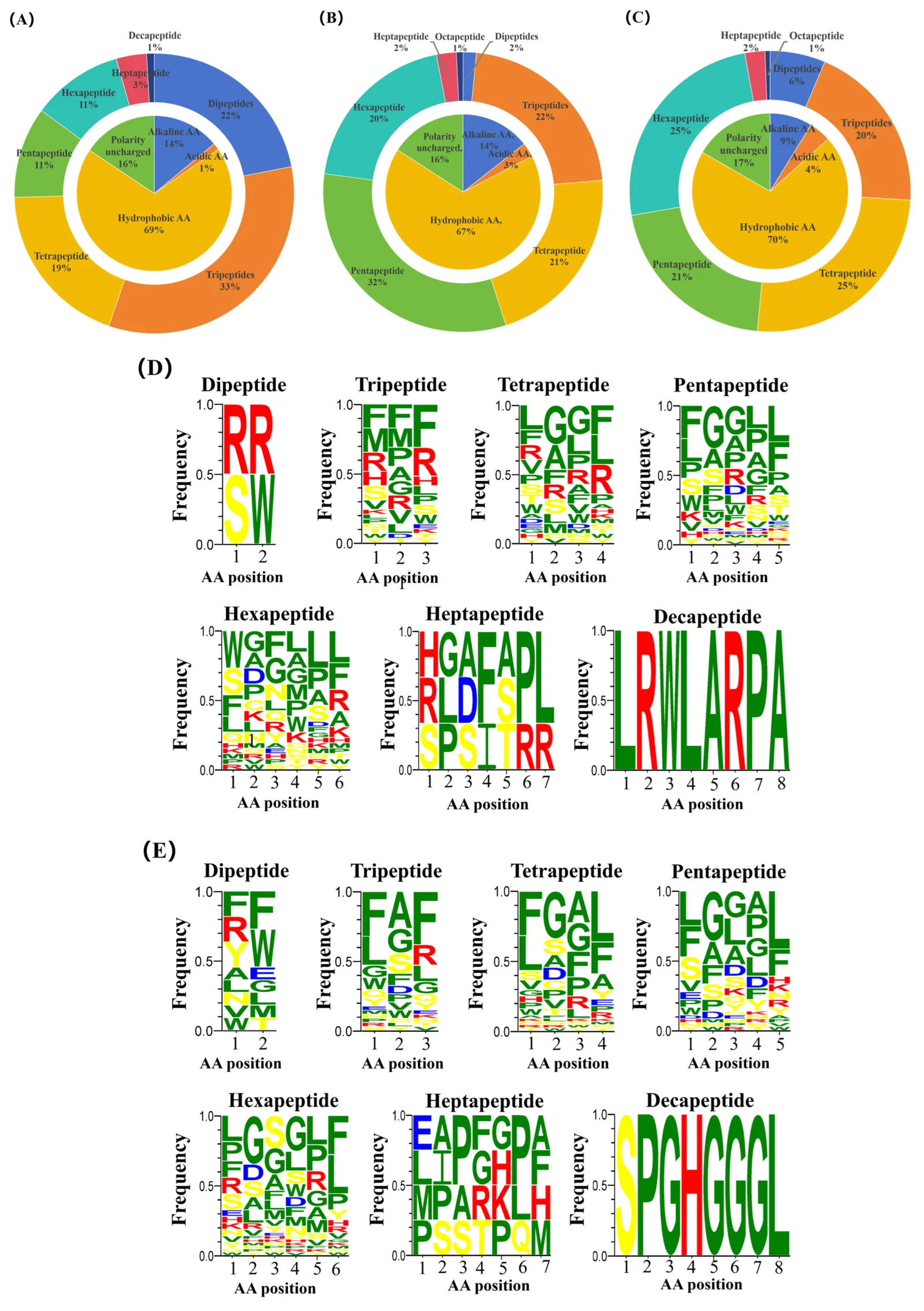
Statistical analysis of peptides with scores > 0.5, including common peptides in RPP-P5 and URPP-P5 (**A**), RPP-P5-specific peptides (**B**), and URPP-P5-specific peptides (**C**). Outer rings represent peptide counts, inner rings depict amino acid property distributions, and peptides of varying lengths in RPP-P5 (**D**) and URPP-P5 (**E**) are characterized for amino acid composition. In D and E, amino acids are colored as follows: green, nonpolar amino acids; blue, acidic amino acids; red, basic amino acids; and yellow, polar amino acids.

Peptide sequences from RPP-P5 and URPP-P5 were identified by UPLC-Q-TOF-MS/MS and analyzed using the Mascot database, yielding 844 and 1036 peptides, respectively. Biological activity scores were predicted using the PeptideRanker database, and peptides with scores above 0.5 were selected for further analysis. Following score-based screening, 114 common peptides were identified in both RPP-P5 and URPP-P5. Additionally, 131 and 173 unique high-activity peptides (score > 0.5) were detected in RPP-P5 and URPP-P5, respectively. The shared peptides mainly consisted of dipeptides to hexapeptides, whereas the unique peptides in both groups were mainly tripeptides to hexapeptides (Figure 3A–C). Analysis of N-terminal residues in the shared peptides revealed 75 hydrophobic amino acids, primarily leucine (Leu, 28 occurrences) and phenylalanine (Phe, 19 occurrences); 17 basic amino acids, including arginine (Arg, 9), lysine (Lys, 5), and histidine (His, 3); and 19 polar uncharged residues, with serine (Ser) appearing 12 times. At the C-terminus, hydrophobic amino acids accounted for 61.40%, mainly Phe, Leu, and methionine (Met), while basic amino acids accounted for 21.93% (25 residues), primarily Arg. Polar uncharged residues represented numbered (15 residues), dominated by tyrosine (Tyr); and acidic residues accounted for 2.63%.

Differences in peptide length distribution and amino acid composition between RPP-P5 and URPP-P5 were further observed (Figure 3D,E). The proportion of basic amino acids in URPP-P5 decreased from 14% to 9% in RPP-P5, and the proportion of pentapeptides decreased from 32% to 21%. Conversely, the combined proportion of dipeptides, tripeptides, and tetrapeptides increased from 45% to 51%. Hydrophobic amino acids at both the N- and C-termini exceeded 60% in both samples, with URPP-P5 exhibiting 70% compared to 67% in RPP-P5, indicating that ultrasonic treatment increased the abundance of hydrophobic residues. Notable differences in terminal amino acid composition were also observed. At the N-terminus, RPP-P5 was dominated by Phe (22), Leu (16), Trp (15), Ser (17), and Arg (10), whereas URPP-P5 showed higher frequencies of Phe (40), Leu (37), Pro (12), and Ser (15). At the C-terminus, RPP-P5 primarily contained Phe (29), Leu (27), and Arg (19), while URPP-P5 displayed Phe (46), Leu (48), Tyr (10), and Arg (11). These changes in peptide length distribution, amino acid composition, and terminal residue characteristics induced by ultrasonic treatment may contribute to variations in peptide biological activity [31].

### 3.6. Antioxidant Activity and Physicochemical Properties of Rice Peptides

The antioxidant capacity of peptides is closely related to their amino acid composition. Peptide sequences enriched in aromatic residues (e.g., Tyr and Trp) and hydrophobic residues (e.g., Gly, Pro, Leu, and Phe) generally exhibit stronger free radical scavenging activity [32,33]. Reduced steric hindrance is also considered to facilitate interactions between peptides and oxidants, thereby enhancing reaction efficiency [33,34]. Based on amino acid composition, predicted bioactivity scores (PeptideRanker), and physicochemical properties, 10 candidate antioxidant peptides were selected from the unique peptides of RPP-P5 and URPP-P5 (Table 3). Peptides identified from RPP-P5 (WGWAF, WGRF, MRF, WGGWLR, RPF, HMFGF, VFF, MCFLPL, SGF, and WGRLL) contained 3–6 amino acids with molecular weights ranging from 310.14 to 774.40 Da. In contrast, peptides from URPP-P5 (WPF, FGF, WWYF, LFF, GGF, WL, RF, FGAF, LW, and FPPP) consisted of 2–4 amino acids, with molecular weights ranging from 279.12 to 700.30 Da. These peptides were enriched in residues such as W, F, L, Y, A, G, R, and P, and exhibited hydrophobicity values from −0.63 to 0.31, steric hindrance between 0.44 and 0.72, and amphiphilicity ranging from 0 to 1.03.

Searches against the BIOPEP database indicated that 18 peptides, except FGF and LW, corresponded to previously unreported antioxidant sequences. Further searches against the NCBI database showed that peptides WGRF, MRF, RPF, VFF, and SGF from RPP-P5, as well as WPF, FGF, LFF, GGF, WL, RF, FGAF, LW, and FPPP from URPP-P5, have not been reported in the literature, suggesting their novelty. Among these candidates, eight peptides (WGWAF, WGRF, WGGWLR, WGRLL, WPF, WWYF, WL, and LW) exhibited TE values exceeding 1.0 μmol TE/μmol in both ABTS and ORAC assays, surpassing the activity of the positive control Trolox. Previous studies have reported that seven oat protein-derived peptides (FNDRLRQGQLL, GLVYIL, GQTV, GQTVFNDRLRQGQLL, YHNAP, YHNAPGLVYIL, and DVNNNANQLEPR) exhibited ORAC values of 0.14–0.67 μmol TE/μmol, while three pea-derived peptides (YLVN, EEHLCFR, and TFY) showed values of 0.37–1.12 μmol TE/μmol. In comparison, several peptides identified in the present study exhibited higher ORAC values, indicating strong oxygen radical scavenging capacity [35,36]. Notably, the novel peptide WGRF from RPP-P5 showed particularly strong antioxidant activity. In addition, three peptides from URPP-P5 (WPF, WL, and LW) also exhibited higher activity than the positive control.

### 3.7. Analysis of Reactive Sites in Synthetic Peptides via Quantum Chemical Calculations

**Figure 4:**
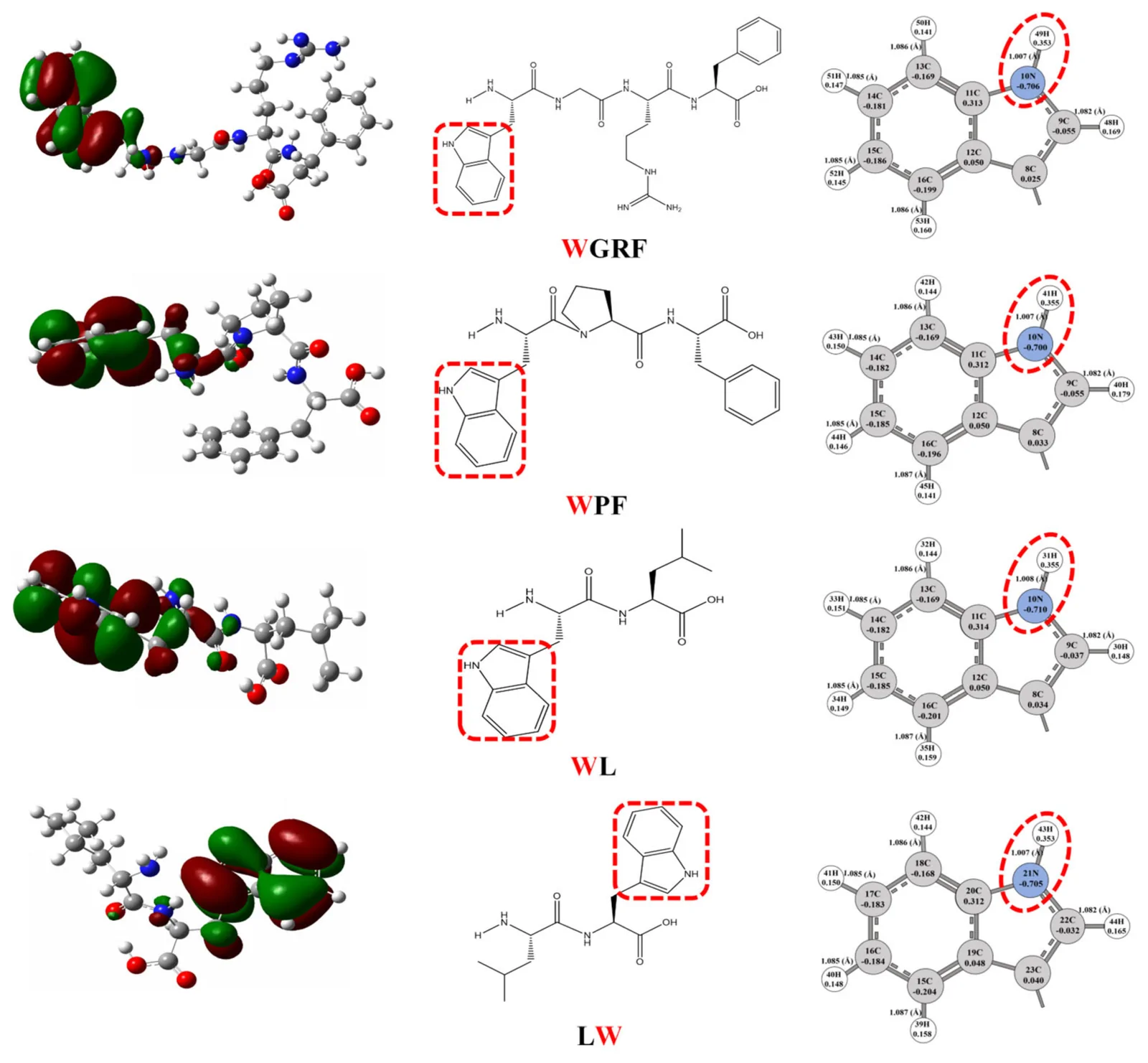
Active-site analysis of the four peptides (WGRF, WPF, WL, and LW) by HOMO and Mulliken charge distribution, and bond length. The red and green regions represent the positive and negative phases of the molecular orbital wavefunction, respectively, while the size and extent of the electron cloud reflect the spatial distribution of electrons.

Quantum chemical calculations were performed to elucidate the relationship between peptide structure and antioxidant activity. The electron-donating capacity of a molecule is closely associated with its antioxidant efficacy. According to frontier molecular orbital theory, a higher EHOMO generally indicates a stronger electron-donating ability. The HOMO, as the frontier orbital, is often considered the primary site for reactions with nucleophiles, particularly hydrogen- and electron-accepting radicals [37]. The HOMO distributions of the four novel peptides are shown in Figure 4 and are predominantly localized on the indole moiety of tryptophan residues. These regions likely represent the most probable electron-donating sites during radical scavenging. Previous studies have demonstrated that antioxidant activity is closely related to specific HOMO-localized regions, typically characterized by relatively negative atomic charges and elongated bond lengths, which facilitate hydrogen atom donation. The mulliken atomic charge distributions and bond lengths within the HOMO regions are summarized in Table S2. The four peptides exhibit similar charge patterns, with negative charges primarily located on nitrogen atoms of the tryptophan indole ring. The proposed active sites correspond to the N–H positions of 31N–71H (WGWAF), 10N–49H (WGRF), 35N–81H (WGGWLR), 10N–54H (WGRLL), 10N–41H (WPF), 10N–60H (WWYF), 10N–31H (WL), and 21N–43H (LW). These N–H groups are considered potential hydrogen donors contributing to radical scavenging activity.

These sites may contribute to peptide antioxidant activity by serving as potential hydrogen-donating sites during free radical scavenging. Notably, the indole N–H bond of tryptophan was consistently predicted as a potential reactive site, regardless of its position at the N-terminus, C-terminus, or within the peptide sequence, suggesting that this structural feature may be associated with the antioxidant properties of tryptophan-containing peptides. Nevertheless, the DFT calculations provide only theoretical evidence based on molecular electronic structure and do not, by themselves, establish the indole N–H bond as a key determinant of antioxidant activity. Its specific contribution therefore requires further validation through structure–activity relationship analyses and experimental approaches.

### 3.8. Investigation of the Mechanism of Antioxidant Peptides

#### 3.8.1. Effects of Antioxidant Peptides on ROS, MDA, GSH, SOD, IL-6, IL-1β, and TNF-α in HaCaT Cells

**Figure 5:**
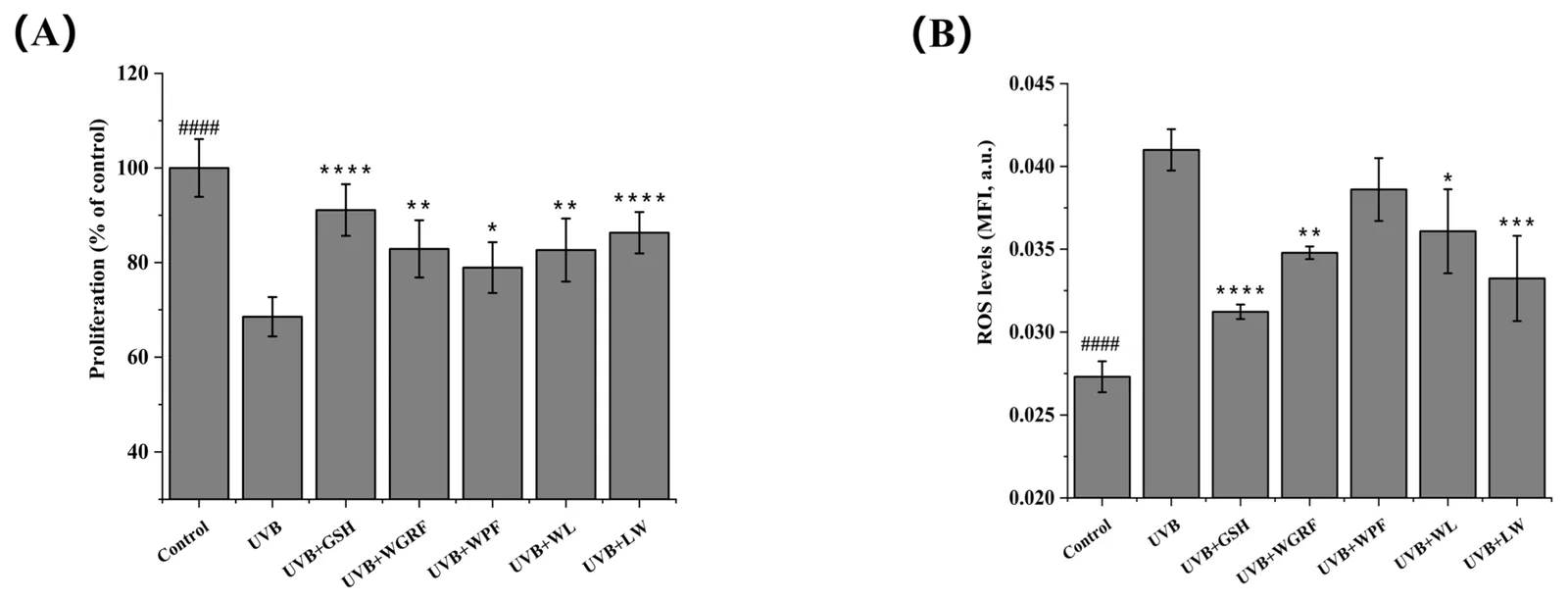

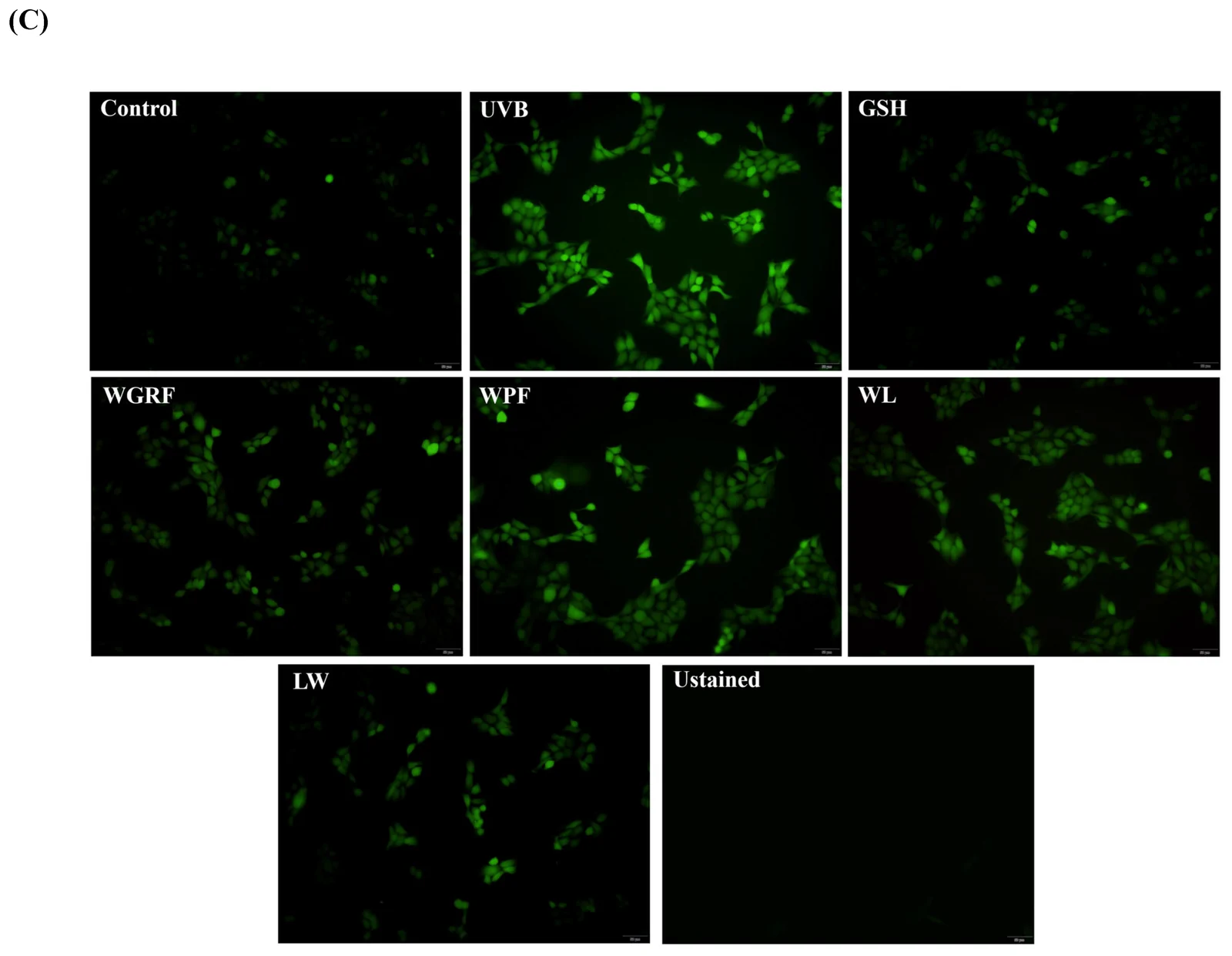
Effects of rice bran peptides on UVB-induced damage in HaCaT cells. Cell viability (**A**), quantitative analysis of fluorescence intensity (**B**), and fluorescence-based visualization of intracellular ROS levels (**C**). Data are presented as the mean ± SD of three independent experiments (*n* = 3). #### indicates a significant difference between the Control and UVB groups (*p* < 0.0001); * indicates a significant difference between the peptide-treated and UVB groups (*p* < 0.05), ** indicates *p* < 0.01, *** indicates *p* < 0.001, and **** indicates *p* < 0.0001.

The cytotoxicity of the peptides was first evaluated in HaCaT cells following 24 h of treatment using the MTT assay. As shown in Figure S1, none of the peptides significantly affected cell viability at concentrations of 0.01–100 μM, with viability remaining within 98.13–108.92% of that of the control group. As shown in Figure 5A, UVB irradiation significantly reduced HaCaT cell viability compared with the control group, indicating successful establishment of the UVB-induced injury model. In peptide-treated groups, cell viability remained at 79–87%, which was significantly higher than that of the UVB model group. No significant cytotoxicity was observed following peptide treatment, suggesting good biocompatibility for subsequent cellular assays.

**Figure 6:**
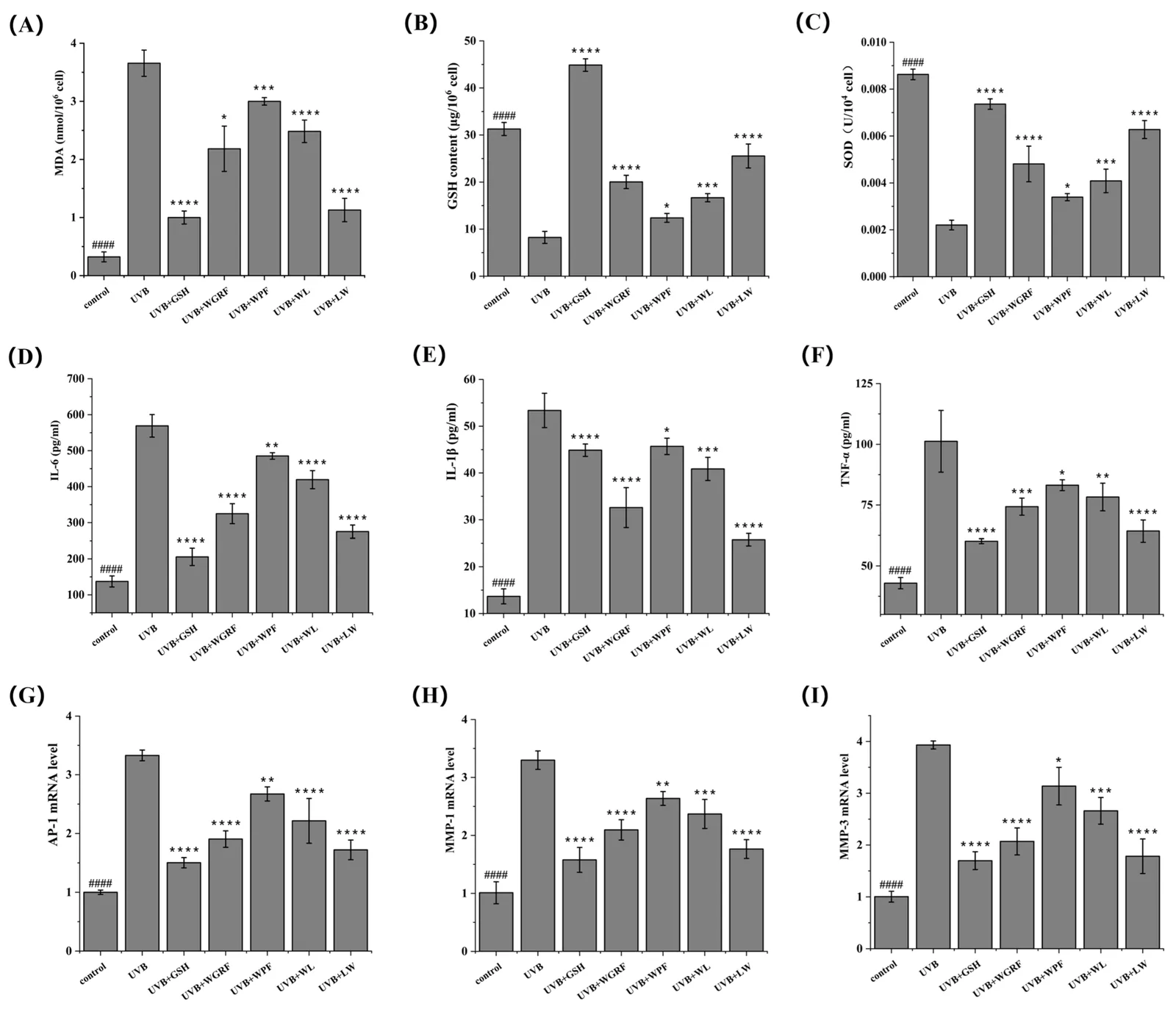

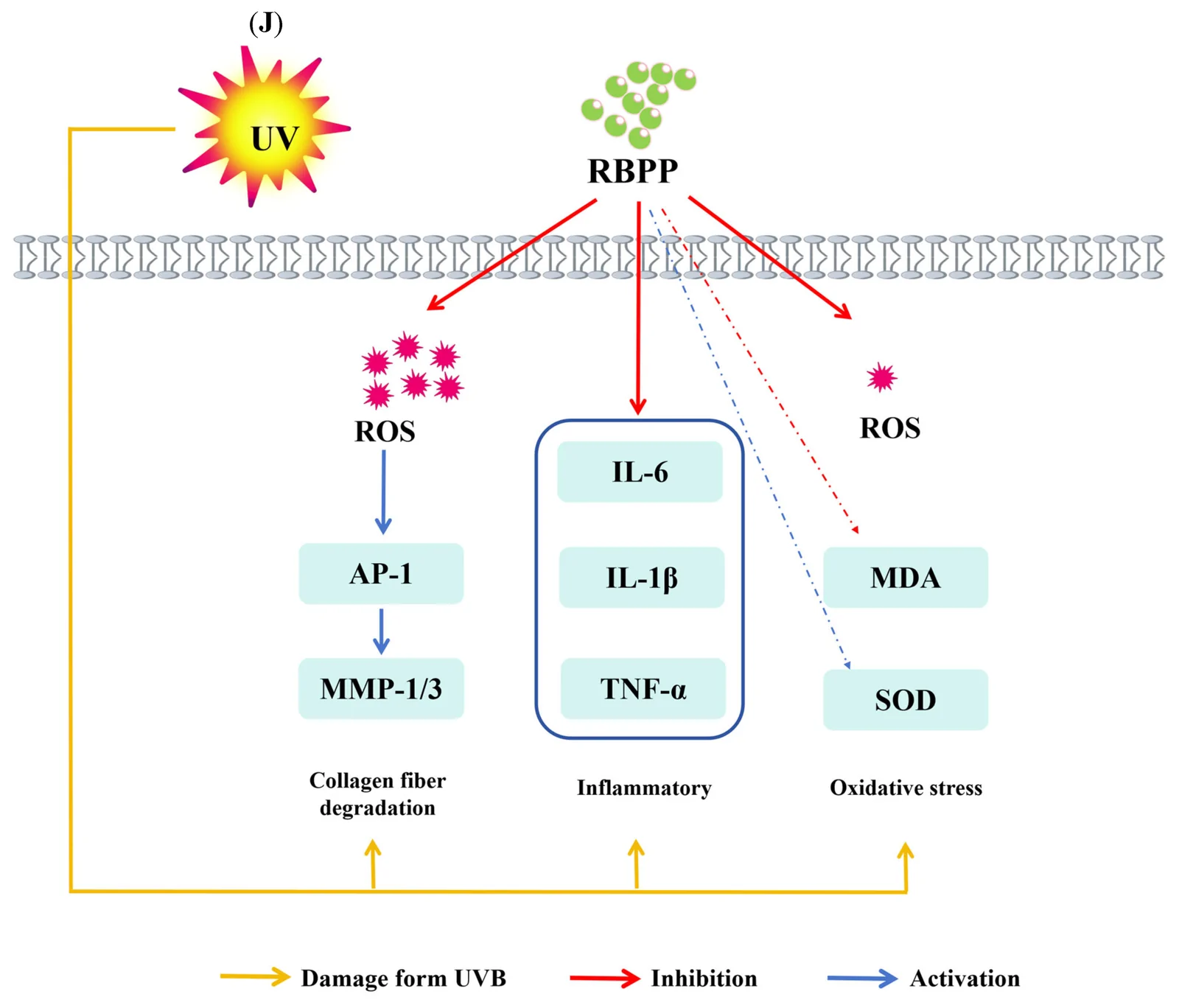
Effects of antioxidant peptides on cellular indicators in HaCaT cells (**A**–**I**), including MDA (**A**), GSH (**B**), SOD (**C**), IL-6 (**D**), IL-1β (**E**), TNF-α (**F**), AP-1 (**G**), MMP-1 (**H**), and MMP-3 (**I**). A potential mechanism by which antioxidant peptides mitigate UVB-induced oxidative damage in HaCaT cells is proposed (**J**). Data are presented as the mean ± SD of three independent experiments (*n* = 3). #### indicates a significant difference between the Control and UVB groups (*p* < 0.0001); * indicates a significant difference between the peptide-treated and UVB groups (*p* < 0.05), ** indicates *p* < 0.01, *** indicates *p* < 0.001, and **** indicates *p* < 0.0001. Yellow arrows indicate UVB-induced damage or promotion; red arrows indicate inhibition or downregulation; blue arrows indicate activation or upregulation.

UVB-induced excessive ROS accumulation leads to oxidative stress in skin cells. Intracellular ROS levels were measured using the DCFH-DA probe, which is converted to fluorescent DCF [38]. As shown in Figure 5B,C, fluorescence intensity in the model group was 1.50 ± 0.05 times that of the control group (*p* < 0.0001), confirming elevated ROS levels. Treatment with antioxidant peptides (WGRF, WL, and LW) significantly reduced ROS levels to 81.07–94.17% of the model group, while the GSH positive control group reached 76.15%. MDA, a product of lipid peroxidation, serves as a marker of oxidative damage. As shown in Figure 6A, MDA levels were significantly decreased in peptide-treated groups compared with the model group, indicating attenuation of UVB-induced oxidative damage. Intracellular GSH content decreased markedly after UVB exposure (from 31.28 ± 1.38 to 8.24 ± 1.27 μg/10^6^ cells), whereas peptide treatment significantly restored GSH levels in Figure 6B. Among them, WGRF and LW showed the strongest effects, reaching 20.03 ± 1.40 and 25.53 ± 2.54 μg/10^6^ cells, respectively, while WPF and WL exhibited moderate recovery. SOD activity, representing enzymatic antioxidant defense, was significantly reduced to 0.25 ± 0.02 times that of the control group after UVB irradiation (Figure 6C *p* < 0.0001). Peptide treatment restored SOD activity to varying extents, with the LW group showing the highest activity (2.85 ± 0.17 times the model group), followed by WGRF, WPF, and WL. Overall, antioxidant peptides enhanced both non-enzymatic and enzymatic antioxidant systems, thereby reducing intracellular ROS levels and alleviating oxidative stress.

UVB irradiation also induced the production of proinflammatory cytokines, including IL-6, IL-1β, and TNF-α. Their levels serve as key indicators of cellular inflammatory status, promoting cell necrosis and apoptosis [39]. As shown in Figure 6D–F, their levels were significantly elevated in the model group (*p* < 0.0001). Peptide treatment markedly reduced the secretion of these cytokines, indicating effective suppression of UVB-induced inflammatory responses. Among all groups, LW exhibited the strongest anti-inflammatory effect, reducing IL-6, IL-1β, and TNF-α levels to 48.35% ± 3.20%, 48.27% ± 2.49%, and 63.49% ± 4.49% of the model group, respectively.

#### 3.8.2. Effects of Antioxidant Peptides on AP-1, MMP-1, and MMP-3 mRNA Expression in Photoaged HaCaT Cells

UVB irradiation activates the transcription factor AP-1, which subsequently upregulates matrix metalloproteinases (MMPs), leading to degradation of extracellular matrix proteins such as collagen and ultimately accelerating skin photoaging. Among these enzymes, MMP-1 and MMP-3 are key mediators of UVB-induced photoaging. Inhibition of AP-1 and its downstream targets (MMP-1 and MMP-3) is therefore commonly used as an indicator of anti-photoaging activity. The mRNA expression levels of AP-1, MMP-1, and MMP-3 were evaluated following peptide treatment. As shown in Figure 6G–I, UVB irradiation significantly upregulated their expression in HaCaT cells compared with the control group (*p* < 0.001), reaching 3.33 ± 0.09, 3.26 ± 0.16, and 3.92 ± 0.08 times of the control levels, respectively. Treatment with antioxidant peptides markedly reduced the expression of these genes to 45.38–80.29% of the UVB group. Among all treatments, the LW group exhibited the strongest inhibitory effect, decreasing AP-1, MMP-1, and MMP-3 expression to 0.52 ± 0.05, 0.54 ± 0.05, and 0.45 ± 0.08 times that of the UVB group, respectively. These results indicate that rice bran-derived antioxidant peptides attenuate the UVB-induced upregulation of AP-1, MMP-1, and MMP-3 mRNA expression, suggesting that they may modulate the AP-1/MMP-associated transcriptional response involved in UVB-induced photodamage. Pearson correlation analysis further demonstrated that intracellular ROS levels were significantly positively correlated (*p* < 0.01) with MDA content, proinflammatory cytokines (IL-6, IL-1β, TNF-α), and metalloproteinase-related mRNA expression (AP-1, MMP-1, MMP-3), while showing significant negative correlations with GSH content and SOD activity (*p* < 0.01). These findings suggest that the anti-photoaging effects of rice bran antioxidant peptides are closely associated with reduced oxidative stress, attenuation of inflammatory responses, accompanied by reduced mRNA expression of AP-1, MMP-1, and MMP-3.

#### 3.8.3. Mechanistic Study of Antioxidant Peptides Inhibiting UVB-Induced Oxidative Damage in HaCaT Cells

As illustrated in Figure 6J, UVB irradiation induces excessive generation of ROS in HaCaT cells. These ROS react with unsaturated fatty acids in cell membranes, leading to enhanced lipid peroxidation and a marked increase in MDA levels. Antioxidant peptides can reduce intracellular ROS levels by scavenging UVB-induced free radicals. GSH, a major non-enzymatic antioxidant, acts synergistically with enzymatic antioxidant systems, such as SOD, to maintain cellular redox homeostasis. However, prolonged oxidative stress disrupts this balance and subsequently triggers inflammatory responses. Rice bran-derived antioxidant peptides were found to suppress UVB-induced inflammation by reducing the production of IL-6, IL-1β, and TNF-α, thereby alleviating inflammatory damage associated with photoaging. Furthermore, proinflammatory cytokines can promote the expression of matrix metalloproteinases (MMPs), accelerating extracellular matrix degradation. By downregulating the UVB-induced expression of AP-1, MMP-1, and MMP-3 mRNA, rice bran antioxidant peptides may modulate AP-1/MMP-related transcriptional responses and alleviate UVB-induced cellular damage [40]. Overall, these results suggest that rice bran antioxidant peptides may alleviate UVB-induced photoaging by attenuating oxidative stress, inflammatory responses, and matrix metalloproteinase mRNA expression, providing a theoretical basis for their potential application in skin protection.

## 4. Conclusions

Four extraction methods significantly influenced the structural properties of rice bran protein and the antioxidant activity of its hydrolysates. Among them, URP exhibited higher purity, increased protein recovery, and a more porous structure, together with enhanced hydration capacity and enzymatic reactivity. The resulting hydrolysate (URPP) showed strong antioxidant activity. The four selected novel peptides exhibited higher antioxidant activities than the positive control and showed potential protective effects in the UVB-induced HaCaT cell photoaging model. These effects were accompanied by reduced oxidative stress and inflammatory responses and modulation of photoaging-related molecular markers. Overall, the findings indicate that the extraction strategy influenced the structural and enzymatic properties of rice bran protein and the antioxidant activity of its derived peptides. These results support the potential of rice bran protein as a source of functional peptides. Further in vivo and clinical studies are required to validate these biological activities and potential applications.

## Figures and Tables

**Table 1:** Basic chemical composition of protein samples obtained by different extraction methods. Data are presented as the mean ± SD of three independent experiments (*n* = 3). Data with different letters in the same test indicate significant differences (*p* < 0.05).

Sample No.	Moisture Content (%)	Starch Content (%)	Protein Content (%)	Fat Content (%)	Fiber Content (%)	Ash Content (%)	Protein Extraction Yield (%)
Rice Bran	9.73 ± 0.06 ^a^	35.93 ± 0.79 ^a^	12.14 ± 0.32 ^e^	11.22 ± 0.30 ^a^	5.68 ± 0.15 ^b^	6.21 ± 0.11 ^e^	-
RP	3.08 ± 0.04 ^b^	3.20 ± 0.06 ^c^	65.83 ± 0.13 ^a^	7.08 ± 0.11 ^d^	4.92 ± 0.03 ^d^	8.76 ± 0.13 ^b^	37.34 ± 0.07 ^d^
URP	2.92 ± 0.03 ^c^	2.56 ± 0.09 ^c^	59.17 ± 0.12 ^b^	10.02 ± 0.06 ^c^	5.27 ± 0.06 ^c^	7.48 ± 0.27 ^d^	47.61 ± 0.10 ^c^
ERP	2.31 ± 0.00 ^d^	16.67 ± 0.17 ^b^	43.25 ± 0.19 ^c^	6.53 ± 0.22 ^e^	3.38 ± 0.02 ^e^	7.86 ± 0.05 ^c^	49.61 ± 0.22 ^b^
UERP	1.85 ± 0.00 ^e^	16.92 ± 0.22 ^b^	37.83 ± 0.27 ^d^	10.50 ± 0.02 ^b^	6.34 ± 0.02 ^a^	10.92 ± 0.04 ^a^	50.70 ± 0.35 ^a^

**Table 2:** Protein content, antioxidant activity, and degree of hydrolysis of protein hydrolysates prepared from protein samples obtained by different extraction methods. Antioxidant activity is expressed as μmol Trolox equivalents per gram of sample (μmol TE/g sample). Data are presented as the mean ± SD of three independent experiments (*n* = 3). Different lowercase letters (a–d) indicate significant differences among groups (*p* < 0.05).

Sample No.	Protein Content (%)	ABTS Assay (μmol TE/g Sample)	ORAC Assay (μmol TE/g Protein)	Hydrolysis Degree (%)
RPP	71.14 ± 2.05 ^b^	646.43 ± 18.33 ^b^	1931.13 ± 13.11 ^b^	22.88 ± 1.03 ^b^
URPP	81.00 ± 1.33 ^a^	684.21 ± 17.67 ^a^	2016.15 ± 6.22 ^a^	33.57 ± 0.46 ^a^
ERPP	51.73 ± 3.14 ^c^	497.37 ± 11.52 ^c^	1818.61 ± 40.11 ^c^	11.16 ± 0.01 ^c^
UERPP	44.38 ± 1.86 ^d^	474.85 ± 12.74 ^c^	1825.62 ± 5.95 ^c^	3.58 ± 0.07 ^d^

**Table 3:** Summary of marks, antioxidant activities, and physicochemical properties of peptides. Antioxidant activity is expressed as μmol Trolox equivalents per gram of sample (μmol TE/μmol). Data are presented as the mean ± SD of three independent experiments (*n* = 3). Different lowercase letters (a–i) indicate significant differences among groups (*p* < 0.05).

Sample No.	Peptide Sequence	Marks	ABTS Assay (μmol TE/μmol)	ORAC Assay (μmol TE/μmol)	Hydrophobicity	Steric Hindrance	Amphipathy
RPP-P5	WGWAF	0.9940	2.1070 ± 0.0088 ^b^	1.8326 ± 0.0337 ^cd^	0.1	0.63	0
	WGRF	0.9921	2.1934 ± 0.0016 ^a^	1.9128 ± 0.0059 ^ab^	−0.42	0.69	0.61
	MRF	0.9837	0.0464 ± 0.0004 ^f^	0.6430 ± 0.0550 ^h^	0.08	0.69	0.32
	WGGWLR	0.9786	2.1957 ± 0.0029 ^a^	1.7733 ± 0.0632 ^d^	−0.18	0.58	0.62
	RPF	0.9778	0.0963 ± 0.0064 ^e^	0.0381 ± 0.0041 ^i^	0.12	0.62	0.25
	HMFGF	0.9772	0.0605 ± 0.0045 ^ef^	0.7542 ± 0.0300 ^g^	−0.39	0.67	0.61
	VFF	0.9693	0.0259 ± 0.0023 ^f^	0.0670 ± 0.0002 ^i^	0.24	0.59	0
	MCFLPL	0.9652	1.5850 ± 0.0468 ^d^	0.8990 ± 0.0365 ^f^	−0.62	0.71	0.82
	SGF	0.9475	0.0552 ± 0.0015 ^ef^	0.0717 ± 0.0095 ^i^	0.29	0.62	0
	WGRLL	0.9403	2.1181 ± 0.0167 ^b^	1.8369 ± 0.0141 ^cd^	−0.22	0.64	0.73
URPP-P5	WPF	0.9973	2.1055± 0.0097 ^b^	1.8542 ± 0.0258 ^bc^	0.09	0.56	0
	FGF	0.9966	0.0342 ± 0.0004 ^f^	–	0.31	0.51	0
	WWYF	0.9965	2.1022 ± 0.0231 ^b^	1.5951 ± 0.0601 ^e^	−0.18	0.69	0.25
	LFF	0.9939	0.0302 ± 0.0008 ^f^	0.0278 ± 0.0046 ^i^	0.1	0.61	0.21
	GGF	0.9873	0.0600 ± 0.0008 ^ef^	0.0109 ± 0.0013 ^i^	0.26	0.59	0.25
	WL	0.9871	2.1560 ± 0.0028 ^a^	1.9308 ± 0.0153 ^a^	0.06	0.61	0.25
	RF	0.9866	0.0294 ± 0.0004 ^f^	0.0577 ± 0.0014 ^i^	0.05	0.69	0.42
	FGAF	0.9853	0.0406 ± 0.0011 ^f^	0.0369 ± 0.0019 ^i^	−0.6	0.72	1.03
	LW	0.9852	1.9904 ± 0.0183 ^c^	1.7857 ± 0.0132 ^d^	−0.31	0.59	0.53
	FPPP	0.9851	0.0581 ± 0.0019 ^ef^	0.0596 ± 0.0078 ^i^	0.10	0.44	0

## Data Availability

The original data presented in this study are openly available through the following public repositories and online resources. Peptide bioactivity prediction was performed using PeptideRanker (accessed on 26 August 2025; http://bioware.ucd.ie/~compass/biowareweb/Server_pages/help/peptideranker/help.php). Allergenicity and toxicity predictions were conducted using AllerTOP (accessed on 2 September 2025; https://www.ddg-pharmfac.net/AllerTOP/) and ToxinPred (accessed on 6 September 2025; https://webs.iiitd.edu.in/raghava/toxinpred/), respectively. Gene sequence data were retrieved from the NCBI GenBank database (accessed on 12 December 2025;https://www.ncbi.nlm.nih.gov/genbank/) under the following accession numbers: AP-1 (NM_002228.4), MMP-1 (NM_001145938.2), MMP-3 (NM_002422.5), and GAPDH (NM_001357943.2). ChatGPT (OpenAI, GPT-5.6 Luna) was used to improve the English language of the manuscript (accessed on 18 August 2026; https://chatgpt.com/).

## Supplementary Materials

The following supporting information can be downloaded at https://www.mdpi.com/article/10.3390/antiox15081049/s1, Table S1: Primers sequence used for RT-qPCR analysis in HaCaT cells; Table S2 Mulliken charge distribution and bond lengths of predicted active sites in four novel peptides; Figure S1: Evaluation of HaCaT cell viability following peptide treatment using the MTT assay. Data are presented as the mean ± SD of three independent experiments (n = 3). Different statistical significance levels are indicated by * *p* < 0.05 and ** *p* < 0.01, compared with the control group.

## Abbreviations

The following abbreviations are used in this manuscript:

AAPH	2,2′-Azobis(2-methylpropionamide) dihydrochloride
ABTS	2,2′-Azino-bis(3-ethylbenzothiazoline-6-sulfonic acid)diammonium salt
AP-1	Activator Protein-1
ASAP	Alkali solubilization acid precipitation
DCF	2′,7′-Dichlorofluorescein
DCFH-DA	2′,7′-Dichlorodihydrofluorescein Diacetate
DFT	Density functional theory
DH	Degree of hydrolysis
DMEM	Dulbecco’s modified Eagle’s medium
DPPH	2,2-Diphenyl-1-picrylhydrazyl
DTT	Dithiothreitol
ELISA	Enzyme-linked immunosorbent assay
ERP	Enzyme-assisted Rice Bran Protein
ERPP	Enzyme-assisted Rice Bran Protein Peptides
ESI	Electrospray ionization
FTIR	Fourier transform infrared spectroscopy
GAPDH	Glyceraldehyde-3-phosphate dehydrogenase
GSH	Glutathione
HaCaT	cells Human epidermal keratinocytes
HOMO	Highest Occupied Molecular Orbital
IL-1β	Interleukin-1 beta
IL-6	Interleukin-6
KBr	Potassium bromide
MDA	Malondialdehyde
Met	methionine
MMP-1	Matrix Metalloproteinase-1
MMP-3	Matrix Metalloproteinase-3
MMPs	Matrix metalloproteinases
OPA	o-Phthaldialdehyde
ORAC	Oxygen radical absorbance capacity
PER	Protein efficiency ratio
Phe	Phenylalanine
RP	Rice Bran Protein
RPP	Rice Bran Protein Peptides
ROS	Reactive oxygen species
SDS	Sodium dodecyl sulfate
SEM	Scanning electron microscopy
SOD	Superoxide dismutase
TE	Trolox Equivalent
TEAC	Trolox equivalent antioxidant capacity
TNF-α	Tumor Necrosis Factor-alpha
Trp	Tryptophan
Tyr	Tyrosine
UERP	Ultrasonic- and Enzyme-assisted Rice Bran Protein
UERPP	Ultrasonic- and Enzyme-assisted Rice Bran Protein Peptides
URP	Ultrasonic-assisted Rice Bran Protein
URPP	Ultrasonic-assisted Rice Bran Protein Peptides
UVB	Ultraviolet B
