# Supplementary material for: Rice Bran Proteins Extracted by Different Methods: Structural Properties and Antioxidant and Anti-Photoaging Activities of Their Hydrolysates
Source: Antioxidants (Basel). 2026 Aug 21;15(8):1049. doi: 10.3390/antiox15081049 (PMC13510287; doi:10.3390/antiox15081049)

Rice bran proteins extracted by different methods: structural properties and antioxidant and anti-photoaging activities of their hydrolysates

Xiao Wang [1,+], Hongru Liu [1,+], Wenhui Tian [1], Bingjie Chen [1], Rongshang Wang [1], Songheng Wu [1], Longshen Wang [1], Jinglin Zhang [1], Hui He [1], Chenxia Liu [1], Qiankun Wang [1], Chunfang Wang [1*], Jucai Xu [2*]

[1] *Crop Breeding and Cultivation Research Institution, Research Center for Agricultural Products Preservation and Processing, Shanghai Academy of Agricultural Sciences, Shanghai, China*
[2] *School of Pharmacy and Food Engineering, Wuyi University, Jiangmen 529020, China*
[*] *Corresponding author, Email: fhwcf@zju.edu.cn, xujucai.happy@163.com*
[+] *These authors contributed equally to this work.*

Table S1 Primers sequence used for RT-qPCR analysis in HaCaT cells

Primer name	Forward primer sequence	Reverse primer sequence	Size（bq）	Accession number	Relevant links
AP-1	CATCACCACCACGCCGACC	CCCTGCCACCGAGGCTACCG	196	NM_002228.4	https://www.ncbi.nlm.nih.gov/nuccore/NM_002228.4
MMP-1	CAGGTATTGGAGGGGATG	GACAGGATTTTGGGAACG	236	NM_001145938.2	https://www.ncbi.nlm.nih.gov/nuccore/NM_001145938.2
MMP-3	GGCTGTATGAAGGAGAGG	CAGTGTTGGCTGAGTGAA	246	NM_002422.5	https://www.ncbi.nlm.nih.gov/nuccore/NM_002422.5
GAPDH	GGAGCGAGATCCCTCCAAAAT	GGCTGTTGTCATACTTCTCATGG	197	NM_001357943.2	https://www.ncbi.nlm.nih.gov/nuccore/NM_001357943.2

Table S2 Mulliken charge distribution and bond lengths of predicted active sites in four novel peptides

**Peptide**	**WGRF**		**WPF**		**WL**		**LW**	
**Mulliken charge**	8C	0.025	8C	0.033	8C	0.034	15C	-0.204
	9C	-0.055	9C	-0.055	9C	-0.037	16C	-0.184
	10N	-0.706	10N	-0.700	10N	-0.710	17C	-0.183
	11C	0.313	11C	-0.312	11C	0.314	18C	-0.168
	12C	0.050	12C	0.050	12C	0.050	19C	0.048
	13C	-0.169	13C	-0.169	13C	-0.169	20C	0.312
	14C	-0.181	14C	-0.182	14C	-0.182	21N	-0.705
	15C	-0.186	15C	-0.185	15C	-0.185	22C	-0.032
	16H	-0.199	16C	-0.196	16C	-0.201	23C	0.040
	48H	0.169	40H	0.179	30H	0.148	39H	0.158
	49H	0.353	41H	0.355	31H	0.355	40H	0.148
	50H	0.141	42H	0.144	32H	0.144	41H	0.150
	51H	0.147	43H	0.150	33H	0.151	42H	0.144
	52H	0.145	44H	0.146	34H	0.149	43H	0.353
	53H	0.160	45H	0.141	35H	0.159	44H	0.165
**Bond length (Å)**	9C-48H	0.978	9C-40H	1.082	9C-30H	1.082	15C-39H	1.087
	10N-49H	1.088	10N-41H	1.007	10N-31H	1.008	16C-40H	1.085
	13C-50H	1.085	13C-42H	1.086	13C-32H	1.086	17C-41H	1.086
	14C-51H	1.086	14C-43H	1.085	14C-33H	1.085	18C-42H	1.086
	15C-52H	1.089	15C-44H	1.085	15C-34H	1.085	21N-43H	1.007
	16C-53H	1.095	16C-45H	1.087	16C-35H	1.087	22C-44H	1.082
**Active site**	10N-49H		10N-41H		10N-31H		21N-43H	

Figure S1 Evaluation of HaCaT cell viability following peptide treatment using the MTT assay. Data are presented as the mean ± SD of three independent experiments (n = 3). Different statistical significance levels are indicated by *p < 0.05 and **p < 0.01, compared with the control group.

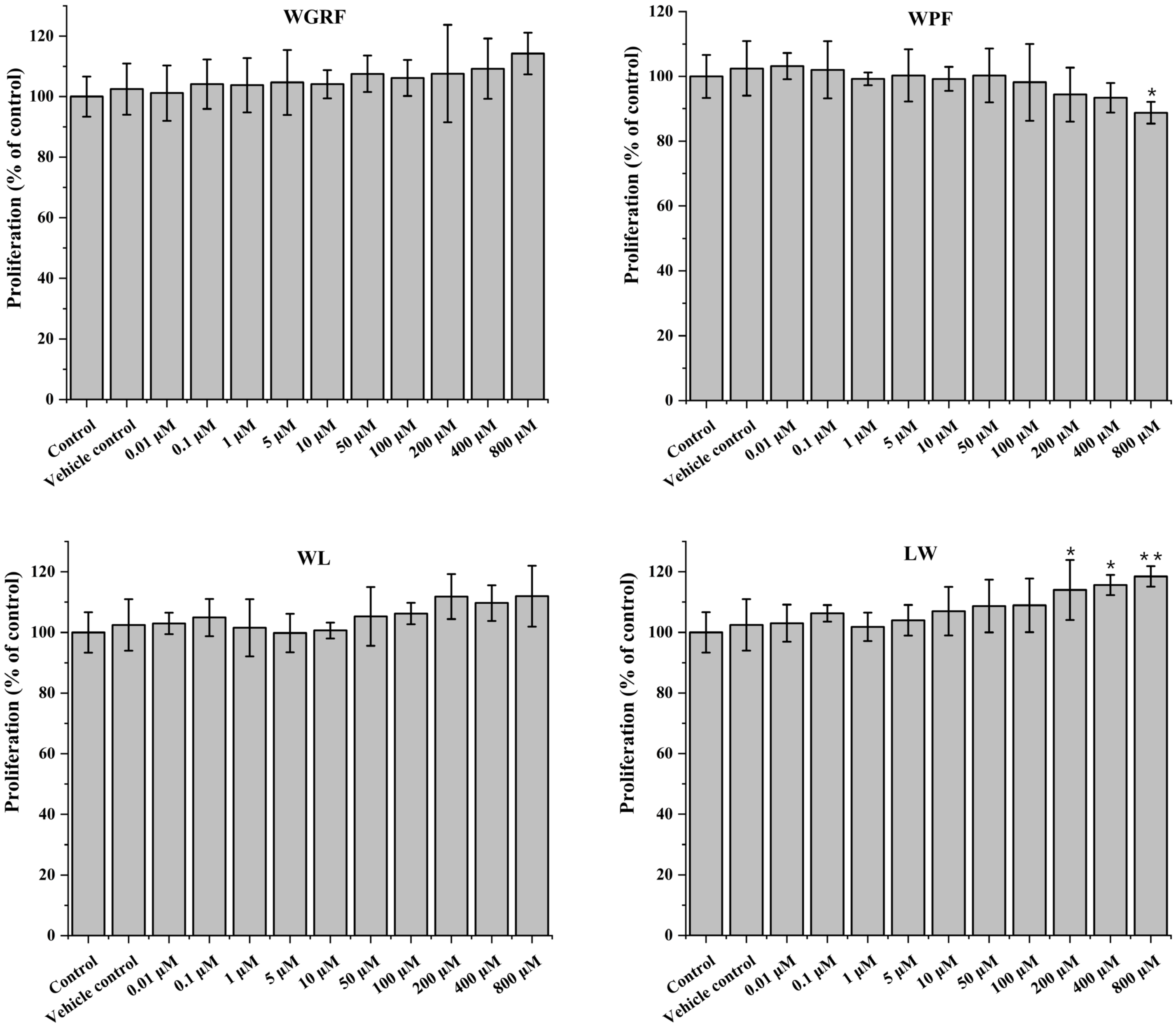

Supplement: Supplementary file 1 [file antioxidants-15-01049-s001.zip › antioxidants-4461076-supplementary.pdf]
